# STARD 2015: an updated list of essential items for reporting diagnostic accuracy studies

**DOI:** 10.1136/bmj.h5527

**Published:** 2015-10-28

**Authors:** Patrick M Bossuyt, Johannes B Reitsma, David E Bruns, Constantine A Gatsonis, Paul P Glasziou, Les Irwig, Jeroen G Lijmer, David Moher, Drummond Rennie, Henrica C W de Vet, Herbert Y Kressel, Nader Rifai, Robert M Golub, Douglas G Altman, Lotty Hooft, Daniël A Korevaar, Jérémie F Cohen

**Affiliations:** 1 Department of Clinical Epidemiology, Biostatistics and Bioinformatics, Academic Medical Centre, University of Amsterdam, Amsterdam, the Netherlands; 2 Julius Center for Health Sciences and Primary Care, University Medical Center Utrecht, University of Utrecht, Utrecht, the Netherlands; 3 Department of Pathology, University of Virginia School of Medicine, Charlottesville, VA, USA; 4 Center for Statistical Sciences, Brown University School of Public Health, Providence, RI, USA; 5 Centre for Research in Evidence-Based Practice, Faculty of Health Sciences and Medicine, Bond University, Gold Coast, Queensland, Australia; 6 Screening and Diagnostic Test Evaluation Program, School of Public Health, University of Sydney, Sydney, New South Wales, Australia; 7 Department of Psychiatry, Onze Lieve Vrouwe Gasthuis, Amsterdam, the Netherlands; 8 Clinical Epidemiology Program, Ottawa Hospital Research Institute, Ottawa, Canada; 9 School of Epidemiology, Public Health and Preventive Medicine, University of Ottawa, Ottawa, Canada; 10 Peer Review Congress, Chicago, IL, USA; 11 Philip R Lee Institute for Health Policy Studies, University of California, San Francisco, CA, USA; 12 Department of Epidemiology and Biostatistics, EMGO Institute for Health and Care Research, VU University Medical Center, Amsterdam, the Netherlands; 13 Department of Radiology, Beth Israel Deaconess Medical Center, Harvard Medical School, Boston, MA, USA; 14 *Radiology* Editorial Office, Boston, MA, USA; 15 Department of Laboratory Medicine, Boston Children’s Hospital, Harvard Medical School, Boston, MA, USA; 16 *Clinical Chemistry* Editorial Office, Washington, DC, USA; 17 Division of General Internal Medicine and Geriatrics and Department of Preventive Medicine, Northwestern University Feinberg School of Medicine, Chicago, IL, USA; 18 *JAMA* Editorial Office, Chicago, IL, USA; 19 Centre for Statistics in Medicine, Nuffield Department of Orthopaedics, Rheumatology and Musculoskeletal Sciences, University of Oxford, Oxford, UK; 20 Dutch Cochrane Centre, Julius Center for Health Sciences and Primary Care, University Medical Center Utrecht, University of Utrecht, Utrecht, the Netherlands; 21 INSERM UMR 1153 and Department of Pediatrics, Necker Hospital, AP-HP, Paris Descartes University, Paris, France.

## Abstract

Incomplete reporting has been identified as a major source of avoidable waste in biomedical research. Essential information is often not provided in study reports, impeding the identification, critical appraisal, and replication of studies. To improve the quality of reporting of diagnostic accuracy studies, the Standards for Reporting Diagnostic Accuracy (STARD) statement was developed. Here we present STARD 2015, an updated list of 30 essential items that should be included in every report of a diagnostic accuracy study. This update incorporates recent evidence about sources of bias and variability in diagnostic accuracy and is intended to facilitate the use of STARD. As such, STARD 2015 may help to improve completeness and transparency in reporting of diagnostic accuracy studies.

As researchers, we talk and write about our studies, not just because we are happy—or disappointed—with the findings, but also to allow others to appreciate the validity of our methods, to enable our colleagues to replicate what we did, and to disclose our findings to clinicians, other health care professionals, and decision makers, all of whom rely on the results of strong research to guide their actions.

Unfortunately, deficiencies in the reporting of research have been highlighted in several areas of clinical medicine.^1^ Essential elements of study methods are often poorly described and sometimes completely omitted, making both critical appraisal and replication difficult, if not impossible. Sometimes study results are selectively reported, and other times researchers cannot resist unwarranted optimism in interpretation of their findings.^2^ ^3^ ^4^ These practices limit the value of the research and any downstream products or activities, such as systematic reviews and clinical practice guidelines.

Reports of studies of medical tests are no exception. A growing number of evaluations have identified deficiencies in the reporting of test accuracy studies.^5^ These are studies in which a test is evaluated against a clinical reference standard, or gold standard; the results are typically reported as estimates of the test’s sensitivity and specificity, which express how good the test is in correctly identifying patients as having the target condition. Other accuracy statistics can be used as well, such as the area under the receiver operating characteristics (ROC) curve or positive and negative predictive values.

Despite their apparent simplicity, such studies are at risk of bias.^6^ ^7^ If not all patients undergoing testing are included in the final analysis, for example, or if only healthy controls are included, the estimates of test accuracy may not reflect the performance of the test in clinical applications. Yet such crucial information is often missing from study reports.

It is now well established that sensitivity and specificity are not fixed test properties. The relative number of false positive and false negative test results varies across settings, depending on how patients present and which tests they have already undergone. Unfortunately, many authors also fail to completely report the clinical context and when, where, and how they identified and recruited eligible study participants.^8^ In addition, sensitivity and specificity estimates can differ because of variable definitions of the reference standard against which the test is being compared. Thus this information should be available in the study report.

## The 2003 STARD statement

To assist in the completeness and transparency of reporting diagnostic accuracy studies, a group of researchers, editors, and other stakeholders developed a minimum list of essential items that should be included in every study report. The guiding principle for developing the list was to select items that, if described, would help readers to judge the potential for bias in the study and appraise the applicability of the study findings and the validity of the authors’ conclusions and recommendations.

The resulting Standards for Reporting Diagnostic Accuracy (STARD) statement appeared in 2003 in two dozen journals.^9^ It was accompanied by editorials and commentaries in several other publications and endorsed by many more.

Since the publication of STARD, several evaluations have pointed to small but statistically significant improvements in reporting accuracy studies (mean gain 1.4 items (95% confidence interval 0.7 to 2.2)).^5^ ^10^ Gradually, more of the essential items are being reported, but the situation remains far from optimal.

## Methods for developing STARD 2015

The STARD steering committee periodically reviews the literature for potentially relevant studies to inform a possible update. In 2013, the steering committee decided that the time was right to update the checklist.

Updating had two major goals: first, to incorporate recent evidence about sources of bias, applicability concerns, and factors facilitating generous interpretation in test accuracy research, and, second, to make the list easier to use. In making modifications, we also considered harmonization with other reporting guidelines, such as Consolidated Standards of Reporting Trials (CONSORT) 2010.^11^

A complete description of the updating process and the justification for the changes are available on the Enhancing the Quality and Transparency of Health Research (EQUATOR) website at www.equator-network.org/reporting-guidelines/stard. In short, we invited the 2003 STARD group members to participate in the updating process, nominate new members, and comment on the general scope of the update. Suggested new members were contacted. As a result, the STARD group has now grown to 85 members that include researchers, editors, journalists, evidence synthesis professionals, funders, and other stakeholders.

STARD group members were then asked to suggest, and later to endorse, proposed changes in a two round, web based survey. This served to prepare a draft list of essential items, which was discussed in the steering committee in a two day meeting in Amsterdam in September 2014. The list was then piloted in different groups: starting and advanced researchers, peer reviewers, and editors.

The general structure of STARD 2015 is similar to that of STARD 2003. A one page document presents 30 items, grouped under sections that follow the introduction, methods, results, and discussion (IMRAD) structure of a scientific article (see table 1). Several of the STARD 2015 items are identical to the ones in the 2003 version. Others have been reworded, combined, or (if complex) split. A few have been added (see table 2 for a summary of new items and table 3 for key terms). A diagram to describe the flow of participants through the study is now expected in all reports (figure[Fig fig1]).

**Table 1 tbl1:** The STARD 2015 list*

Section and topic	No	Item
**Title or abstract**		
	1	Identification as a study of diagnostic accuracy using at least one measure of accuracy (such as sensitivity, specificity, predictive values, or AUC)
**Abstract**		
	2	Structured summary of study design, methods, results, and conclusions (for specific guidance, see STARD for Abstracts)
**Introduction**		
	3	Scientific and clinical background, including the intended use and clinical role of the index test
	4	Study objectives and hypotheses
**Methods**		
Study design	5	Whether data collection was planned before the index test and reference standard were performed (prospective study) or after (retrospective study)
Participants	6	Eligibility criteria
	7	On what basis potentially eligible participants were identified (such as symptoms, results from previous tests, inclusion in registry)
	8	Where and when potentially eligible participants were identified (setting, location, and dates)
	9	Whether participants formed a consecutive, random, or convenience series
Test methods	10a	Index test, in sufficient detail to allow replication
	10b	Reference standard, in sufficient detail to allow replication
	11	Rationale for choosing the reference standard (if alternatives exist)
	12a	Definition of and rationale for test positivity cut-offs or result categories of the index test, distinguishing pre-specified from exploratory
	12b	Definition of and rationale for test positivity cut-offs or result categories of the reference standard, distinguishing pre-specified from exploratory
	13a	Whether clinical information and reference standard results were available to the performers or readers of the index test
	13b	Whether clinical information and index test results were available to the assessors of the reference standard
Analysis	14	Methods for estimating or comparing measures of diagnostic accuracy
	15	How indeterminate index test or reference standard results were handled
	16	How missing data on the index test and reference standard were handled
	17	Any analyses of variability in diagnostic accuracy, distinguishing pre-specified from exploratory
	18	Intended sample size and how it was determined
**Results**		
Participants	19	Flow of participants, using a diagram
	20	Baseline demographic and clinical characteristics of participants
	21a	Distribution of severity of disease in those with the target condition
	21b	Distribution of alternative diagnoses in those without the target condition
	22	Time interval and any clinical interventions between index test and reference standard
Test results	23	Cross tabulation of the index test results (or their distribution) by the results of the reference standard
	24	Estimates of diagnostic accuracy and their precision (such as 95% confidence intervals)
	25	Any adverse events from performing the index test or the reference standard
**Discussion**		
	26	Study limitations, including sources of potential bias, statistical uncertainty, and generalisability
	27	Implications for practice, including the intended use and clinical role of the index test
**Other information**		
	28	Registration number and name of registry
	29	Where the full study protocol can be accessed
	30	Sources of funding and other support; role of funders

*At the start of each item row, authors should specify the page number of the manuscript where the item can be found.

**Table 2 tbl2:** Summary of new items in STARD 2015

No	Item	Rationale
2	Structured abstract	Abstracts are increasingly used to identify key elements of study design and results.
3	Intended use and clinical role of the test	Describing the targeted application of the test helps readers to interpret the implications of reported accuracy estimates.
4	Study hypotheses	Not having a specific study hypothesis may invite generous interpretation of the study results and “spin” in the conclusions.
18	Sample size	Readers want to appreciate the anticipated precision and power of the study and whether authors were successful in recruiting the targeted number of participants.
26-27	Structured discussion	To prevent jumping to unwarranted conclusions, authors are invited to discuss study limitations and draw conclusions keeping in mind the targeted application of the evaluated tests (see item 3).
28	Registration	Prospective test accuracy studies are trials, and, as such, they can be registered in clinical trial registries, such as ClinicalTrials.gov, before their initiation, facilitating identification of their existence and preventing selective reporting.
29	Protocol	The full study protocol, with more information about the predefined study methods, may be available elsewhere, to allow more fine grained critical appraisal.
30	Sources of funding	Awareness of the potentially compromising effects of conflicts of interest between researchers’ obligations to abide by scientific and ethical principles and other goals, such as financial ones; test accuracy studies are no exception.

**Table 3 tbl3:** Key STARD terminology

Term	Explanation
Medical test	Any method for collecting additional information about the current or future health status of a patient
Index test	The test under evaluation
Target condition	The disease or condition that the index test is expected to detect
Clinical reference standard	The best available method for establishing the presence or absence of the target condition; a gold standard would be an error-free reference standard
Sensitivity	Proportion of those with the target condition who test positive with the index test
Specificity	Proportion of those without the target condition who test negative with the index test
Intended use of the test	Whether the index test is used for diagnosis, screening, staging, monitoring, surveillance, prediction, prognosis, or other reasons
Role of the test	The position of the index test relative to other tests for the same condition (for example, triage, replacement, add-on, new test)

**Figure fig1:**
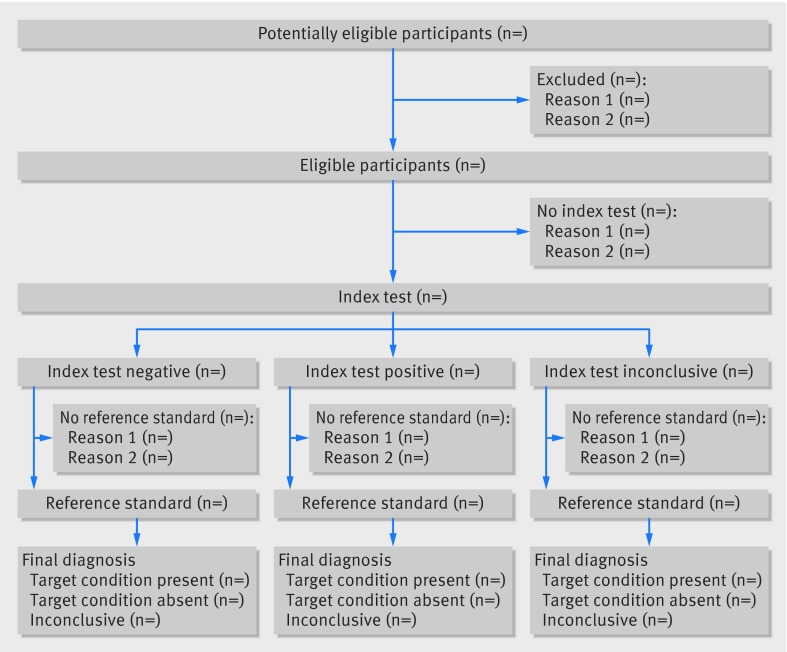
Prototypical STARD diagram to report flow of participants through the study.

## Scope

STARD 2015 replaces the original version published in 2003; those who would like to refer to STARD are invited to cite this article. The list of essential items can be seen as a minimum set, and an informative study report will typically present more information. Yet we hope to find all applicable items in a well prepared report of a diagnostic accuracy study.

Authors are invited to use STARD when preparing their study reports. Reviewers can use the list to verify that all essential information is available in a submitted manuscript and suggest changes if key items are missing.

We trust that journals that endorsed STARD in 2003 or later will recommend the use of this updated version and encourage compliance in submitted manuscripts. We hope that even more journals, and journal organizations, will promote the use of this and comparable reporting guidelines. Funders and research institutions may promote or mandate adherence to STARD as a way to maximize the value of research and downstream products or activities.

STARD may also be beneficial for reporting other studies that evaluate the performance of tests. This includes prognostic studies, which can classify patients on the basis of whether a future event happens; monitoring studies, in which tests are supposed to detect or predict an adverse event or lack of response; studies evaluating treatment selection markers; and more. We and others have found most of the STARD items useful when reporting and examining such studies, although STARD primarily targets diagnostic accuracy studies.

Diagnostic accuracy is not the only expression of test performance, nor is it always the most meaningful.^12^ Incremental accuracy from combining tests, relative to a single test, can be more informative, for example.^13^ For continuous tests, dichotomization into test positives and negatives may not always be indicated. In such cases, the desirable computational and graphical methods for expressing test performance are different, although many of the methodological precautions would be the same, and STARD can help in reporting the study in an informative way. Other reporting guidelines target more specific forms of tests, such as Transparent Reporting of a Multivariable Prediction Model for Individual Prognosis or Diagnosis (TRIPOD) for multivariable prediction models.^14^

Although STARD focuses on full study reports of test accuracy studies, the items can also be helpful when writing conference abstracts, including information in trial registries, and developing protocols for such studies. Additional initiatives are underway to provide more specific guidance for each of these applications.

## STARD extensions and applications

The STARD statement was designed to apply to all types of medical tests. The STARD group believed that a single checklist, for all diagnostic accuracy studies, would be more widely disseminated and more easily accepted by authors, peer reviewers, and journal editors than separate lists for different types of tests such as imaging, biochemistry, or histopathology.

Having a general list may necessitate additional instructions for informative reporting, with more information for specific types of tests, specific applications, or specific forms of analysis. Such guidance could describe the preferred methods for studying and reporting measurement uncertainty, for example, without changing any of the other STARD items. The STARD group welcomes the development of such STARD extensions and invites interested groups to contact the STARD executive committee before developing them.

Other groups may want to develop additional guidance to facilitate the use of STARD for specific applications. An example of such a STARD application was prepared for history taking and physical examination.^15^ Another type of application is the use of STARD for specific target conditions such as dementia.^16^

## Availability

The new STARD 2015 list and all related documents can be found on the STARD pages of the EQUATOR website. EQUATOR is an international initiative that seeks to improve the value of published health research literature by promoting transparent and accurate reporting and wider use of robust reporting guidelines.^17^ ^18^ The STARD group believes that working more closely with EQUATOR and other reporting guideline developers will help us to better reach shared objectives. We have updated the 2003 explanation and elaboration document, which can also be found at the EQUATOR website. This document explains the rationale for each item and gives examples.

The STARD list is released under a Creative Commons license. This allows everyone to use and distribute the work if they acknowledge the source. The STARD statement was originally reported in English, but several groups have worked on translations in other languages. We welcome such translations, which are preferably developed by groups of researchers, by use of a cyclical development process, with back-translation to the original language and user testing.^19^ We have also applied for a trademark for STARD to ensure that the steering committee has the exclusive right to use the word “STARD” to identify goods or services.

## Increasing value, reducing waste

The STARD steering committee is aware that building a list of essential items is not sufficient to achieve substantial improvements in reporting completeness, as the modest improvement after introduction of the 2003 list has shown. We see this list not as the final product, but as the starting point for building more specific instruments to stimulate complete and transparent reporting, such as a checklist and a writing aid for authors, tools for reviewers and editors, instruction videos, and teaching materials, all based on this STARD list of essential items.

Incomplete reporting has been identified as one of the sources of avoidable waste in biomedical research.^1^ Since STARD was initiated, several other initiatives have been undertaken to enhance the reproducibility of research and promote greater transparency.^20^ Multiple factors are at stake, but incomplete reporting is one of them. We hope that this update of STARD, together with additional implementation initiatives, will help authors, editors, reviewers, readers, and decision makers to collect, appraise, and apply the evidence needed to strengthen decisions and recommendations about medical tests. In the end, we are all to benefit from more informative and transparent reporting: as researchers, as healthcare professionals, as payers, and as patients.

## Notes

This article is being simultaneously published in October 2015 by *The BMJ*, *Radiology*, and *Clinical Chemistry*. This article is published under the Creative Commons CC BY-NC license http://creativecommons.org/licenses/by-nc/4.0.

STARD Group collaborators: Todd Alonzo, Douglas G Altman, Augusto Azuara-Blanco, Lucas Bachmann, Jeffrey Blume, Patrick M Bossuyt, Isabelle Boutron, David Bruns, Harry Büller, Frank Buntinx, Sarah Byron, Stephanie Chang, Jérémie F Cohen, Richelle Cooper, Joris de Groot, Henrica C W de Vet, Jon Deeks, Nandini Dendukuri, Jac Dinnes, Kenneth Fleming, Constantine A Gatsonis, Paul P Glasziou, Robert M Golub, Gordon Guyatt, Carl Heneghan, Jørgen Hilden, Lotty Hooft, Rita Horvath, Myriam Hunink, Chris Hyde, John Ioannidis, Les Irwig, Holly Janes, Jos Kleijnen, André Knottnerus, Daniël A Korevaar, Herbert Y Kressel, Stefan Lange, Mariska Leeflang, Jeroen G Lijmer, Sally Lord, Blanca Lumbreras, Petra Macaskill, Erik Magid, Susan Mallett, Matthew McInnes, Barbara McNeil, Matthew McQueen, David Moher, Karel Moons, Katie Morris, Reem Mustafa, Nancy Obuchowski, Eleanor Ochodo, Andrew Onderdonk, John Overbeke, Nitika Pai, Rosanna Peeling, Margaret Pepe, Steffen Petersen, Christopher Price, Philippe Ravaud, Johannes B. Reitsma, Drummond Rennie, Nader Rifai, Anne Rutjes, Holger Schunemann, David Simel, Iveta Simera, Nynke Smidt, Ewout Steyerberg, Sharon Straus, William Summerskill, Yemisi Takwoingi, Matthew Thompson, Ann van de Bruel, Hans van Maanen, Andrew Vickers, Gianni Virgili, Stephen Walter, Wim Weber, Marie Westwood, Penny Whiting, Nancy Wilczynski, Andreas Ziegler.

## Notes

Cite this as: *BMJ* 2015;351:h5527

## References

[1] Glasziou P, Altman DG, Bossuyt P, et al. Reducing waste from incomplete or unusable reports of biomedical research. Lancet 2014;383:267-76. 24411647 10.1016/S0140-6736(13)62228-X

[2] Boutron I, Dutton S, Ravaud P, Altman DG. Reporting and interpretation of randomized controlled trials with statistically nonsignificant results for primary outcomes. JAMA 2010;303:2058-64. 20501928 10.1001/jama.2010.651

[3] Ochodo EA, de Haan MC, Reitsma JB, et al. Overinterpretation and misreporting of diagnostic accuracy studies: evidence of “spin.” Radiology 2013;267:581-8. 23360738 10.1148/radiol.12120527

[4] Mathieu S, Boutron I, Moher D, Altman DG, Ravaud P. Comparison of registered and published primary outcomes in randomized controlled trials. JAMA 2009;302:977-84. 19724045 10.1001/jama.2009.1242

[5] Korevaar DA, Wang J, van Enst WA, et al. Reporting diagnostic accuracy studies: some improvements after 10 years of STARD. Radiology 2015;274:781-9. 25350641 10.1148/radiol.14141160

[6] Lijmer JG, Mol BW, Heisterkamp S, et al. Empirical evidence of design-related bias in studies of diagnostic tests. JAMA 1999;282:1061-6. 10493205 10.1001/jama.282.11.1061

[7] Whiting PF, Rutjes AW, Westwood ME, Mallett S. A systematic review classifies sources of bias and variation in diagnostic test accuracy studies. J Clin Epidemiol 2013;66:1093-104. 23958378 10.1016/j.jclinepi.2013.05.014

[8] Irwig L, Bossuyt P, Glasziou P, Gatsonis C, Lijmer J. Designing studies to ensure that estimates of test accuracy are transferable. BMJ 2002;324:669-71. 11895830 10.1136/bmj.324.7338.669 PMC1122584

[9] Bossuyt PM, Reitsma JB, Bruns DE, et al. Towards complete and accurate reporting of studies of diagnostic accuracy: the STARD Initiative. Radiology 2003;226:24-8. 12511664 10.1148/radiol.2261021292

[10] Korevaar DA, van Enst WA, Spijker R, Bossuyt PM, Hooft L. Reporting quality of diagnostic accuracy studies: a systematic review and meta-analysis of investigations on adherence to STARD. Evid Based Med 2014;19:47-54. 24368333 10.1136/eb-2013-101637

[11] Schulz KF, Altman DG, Moher D, Group C. CONSORT 2010 statement: updated guidelines for reporting parallel group randomised trials. J Clin Epidemiol 2010;63:834-40. 20346629 10.1016/j.jclinepi.2010.02.005

[12] Bossuyt PM, Reitsma JB, Linnet K, Moons KG. Beyond diagnostic accuracy: the clinical utility of diagnostic tests. Clin Chem 2012;58:1636-43. 22730450 10.1373/clinchem.2012.182576

[13] Moons KG, de Groot JA, Linnet K, Reitsma JB, Bossuyt PM. Quantifying the added value of a diagnostic test or marker. Clin Chem 2012;58:1408-17. 22952348 10.1373/clinchem.2012.182550

[14] Collins GS, Reitsma JB, Altman DG, Moons KG. Transparent reporting of a multivariable prediction model for individual prognosis or diagnosis (TRIPOD): the TRIPOD statement. BMJ 2015;350:g7594. 25569120 10.1136/bmj.g7594

[15] Simel DL, Rennie D, Bossuyt PM. The STARD statement for reporting diagnostic accuracy studies: application to the history and physical examination. J Gen Intern Med 2008;23:768-74. 18347878 10.1007/s11606-008-0583-3 PMC2517891

[16] Noel-Storr AH, McCleery JM, Richard E, et al. Reporting standards for studies of diagnostic test accuracy in dementia: the STARDdem Initiative. Neurology 2014;83:364-73. 24944261 10.1212/WNL.0000000000000621 PMC4115600

[17] Altman DG, Simera I, Hoey J, Moher D, Schulz K. EQUATOR: reporting guidelines for health research. Lancet 2008;371:1149-50. 18395566 10.1016/S0140-6736(08)60505-X

[18] Simera I, Moher D, Hirst A, et al. Transparent and accurate reporting increases reliability, utility, and impact of your research: reporting guidelines and the EQUATOR Network. BMC Med 2010;8:24. 20420659 10.1186/1741-7015-8-24 PMC2874506

[19] Beaton DE, Bombardier C, Guillemin F, Ferraz MB. Guidelines for the process of cross-cultural adaptation of self-report measures. Spine 2000;25:3186-91. 11124735 10.1097/00007632-200012150-00014

[20] Collins FS, Tabak LA. Policy: NIH plans to enhance reproducibility. Nature 2014;505:612-3. 24482835 10.1038/505612a PMC4058759

